# Books Reviewed

**Published:** 1865-06

**Authors:** 


*A Vest-Pocket Medical Lexicon: being a Dictionary of Words, Terms, and Symbols of Medical Science, collated from the best authorities, with the addition of new words not before introduced into a Lexicon, with an Appendix.* By D. B. St. John Roosa, M. D., *Aural Surgeon to the New York Eye and Ear Infirmary. New York:* Wm. Wood & Co., 61 *Walker Street*, 1865.

This is a miniature Medical Lexicon, being only about three inches long and one inch thick, containing 270 pages; designed to serve as a "vest-pocket" companion to the student while attending medical lectures; and is very convenient to any one wishing a ready reference dictionary. It contains also abbreviations and symbols used in writing prescriptions, examples of prescriptions, poisons and their antidotes, and a table of elementary substances with their symbols and equivalents.

---

*Transactions of the Medical Society of the State of Pennsylvania, at its Fifteenth Annual Session, held in Philadelphia*, 1864.

We have received a copy of the Transactions of the above Medical Society. It reports cerebro-spinal meningitis to have prevailed epidemically in seven counties. Diphtheria was reported in nine, typhoid fever in seven, measles in seven, scarlatina in six, variola in five, erysipelas in five, and whooping-cough in four. The President, Dr. Wilson Jewell, delivered the Annual Address. Dr. Andrew Nebinger, on behalf of the Committee of Arrangements of the Philadelphia County Medical Society, welcomed the delegates in an elegant and appropriate speech. No medical papers were read before the Society. The volume consists mainly of the reports of the various County Societies of the State.

*Fourteenth Anniversary Meeting of the Illinois State Medical Society, held in Chicago, May* 3, 4 *and* 5, 1864.

The Medical Society of the State of Illinois held their Fourteenth Annual Meeting in Chicago, May, 1864. In the absence of the President, Dr. A. H. Luce, first Vice President occupied the chair. Dr. M. O. Heydock of Chicago, Chairman of the Committee of Arrangements, welcomed the members in a brief and appropriata address. The Committee on Practical Medicine reported erysipelas and cerebro-spinal meningitis to have prevailed epidemically. Also such improvements as had been made in the practice of medicine during the preceding year. The Committee on the Diseases of the Eye, found as the result of their investigations, that diseases of the conjunctiva formed by far a greater class than those of any other part of the eye. The Committee on Orthopedic Surgery reported a large number of interesting and important cases of the various kinds of talipes, and instruments for their treatment. There are reports from the Committee on Surgery, and articles on puerperal fever and cerebro-spinal meningitis, which the want of space prevents further notice.

